# Effect of Intramuscularly Administered Oxytetracycline or Enrofloxacin on Vancomycin-Resistant Enterococci, Extended Spectrum Beta-Lactamase- and Carbapenemase-Producing *Enterobacteriaceae* in Pigs

**DOI:** 10.3390/ani12050622

**Published:** 2022-03-01

**Authors:** Elena González-Fandos, Alba Martínez-Laorden, Ana Abad-Fau, Eloisa Sevilla, Rosa Bolea, María Jesús Serrano, Olga Mitjana, Cristina Bonastre, Alicia Laborda, María Victoria Falceto, Rafael Pagán

**Affiliations:** 1 Department of Food Technology, CIVA Research Center, University of La Rioja, 26006 Logrono, Spain; alba.mar.lao@outlook.es; 2 AgriFood Research Institute of Aragón-IA2, University of Zaragoza-CITA, 50013 Zaragoza, Spain; ana-abad-fau@hotmail.com (A.A.-F.); esevillr@unizar.es (E.S.); rbolea@unizar.es (R.B.); mjserran@unizar.es (M.J.S.); omitjana@unizar.es (O.M.); cbonastr@unizar.es (C.B.); alaborda@unizar.es (A.L.); vfalceto@unizar.es (M.V.F.); pagan@unizar.es (R.P.)

**Keywords:** antimicrobial resistance, antibiotic use, swine, ESBL, VRE, carbapenemases, *Enterococcus* spp., *Escherichia coli*, *Enterococcus faecium*, *Enterococcus faecalis*, *Klebsiella pneumoniae*

## Abstract

**Simple Summary:**

Nowadays, there is great concern about the prevalence of multidrug resistant bacteria in food-producing animals since they are potential sources of transmission to humans. The aim of this work was to evaluate the effect of two antibiotics (oxytetracycline and enrofloxacin) treatments in pigs on resistant bacteria that are considered a threat to public health. This study highlights that the use of oxytetracycline or enrofloxacin in food-producing animals could select resistant bacteria in pig faeces. Special care should be taken to avoid faecal contamination of carcasses during slaughter.

**Abstract:**

Nowadays, there is a great concern about the prevalence of multidrug resistant *Enterococcus* spp. and *Enterobacteriaceae* in food-producing animals. The aim of this work was to evaluate the effect of oxytetracycline or enrofloxacin treatment on vancomycin-resistant enterococci (VRE), extended spectrum β-lactamase (ESBL) and carbapenemase-producing *Enterobacteriaceae* in pigs. A total of 26 piglets were received and distributed in three groups. Group 1 was treated with enrofloxacin (N = 12), group 2 with oxytetracycline (N = 10) and group 3 did not receive any treatment (control group) (N = 4). A higher number of vancomycin-resistant *E. faecium* were recovered compared to *E. faecalis.* In the pigs treated with enrofloxacin, vancomycin resistant *E. faecium* was found in a higher percentage of animals than in the control group. ESBL-producing *E. coli* was not detected in rectal samples from control animals. However, it was detected in 17–20% of animals treated with oxytetracycline on days 6 to 17 and in 17–50% of the animals treated with enrofloxacin. Carbapenemase-producing *E. coli* was isolated in animals treated with oxytetracycline, but not in animals treated with enrofloxacin or in the control group. This study highlights that the use of oxytetracycline or enrofloxacin in food-producing animals could select ESBL and carbapenemase-producing *E. coli*. Further studies shall be needed to validate the results obtained, considering a more robust and extended experimental design.

## 1. Introduction

The increase of antimicrobial resistance is considered a great threat to animal and human health, being responsible for a large number of human deaths annually [1,2,3,4]. Antimicrobials are necessary for the treatment of bacterial infections in food-producing animals, but they can contribute to the expansion of antimicrobial resistance [2,5]. The antibiotic resistance problem should be approached in a “One Health” perspective considering human medicine, veterinary medicine and the environment since all living species and the environment are interconnected [6]. Thus, reducing the transmission and dissemination of multidrug resistance in one of these sectors might influence the others [7].

The antimicrobial resistance of bacterial species from food-producing animals could influence human health since they are potential sources of transmission to humans [8,9]. There is a special concern on enteric bacteria from animals, such as *Enterococcus* spp. and *Enterobacteriaceae* [10,11]. Nowadays, the worldwide spread of vancomycin-resistant-enterococci (VRE) along with extended-spectrum β-lactamase (ESBL) and carbapenemase-producing *Enterobacteriaceae* is considered a threat to public health [12,13,14,15].

Enterococci are part of the natural intestinal microbiota of animals and humans. These bacteria are ubiquitous and can be found in water, soil and plants [16]. Enterococci can be spread by indirect contact (faeces, contaminated food) as well as direct contact with animals [16,17]. The *Enterococcus* species of concern to human health are *Enterococcus faecalis* and *Enterococcus faecium*. On the one hand, one of the primary factors contributing to the morbidity related to infections caused by *Enterococcus* is their antimicrobial resistance [18]. Moreover, it is of concern the ability of enterococci to acquire resistance to antibiotics and their possible role as a reservoir of antibiotic resistance genes that could be transferred to other bacteria. Since vancomycin is one of the treatments of choice for severe infections caused by multi-drug-resistant enterococci in humans, it is of special worry the presence of VRE in animals [19]. Vancomycin is not used in food-producing animals as it pertains to group A of the EMA (European Medicines Agency) categorisation of antibiotics used in Veterinary Medicine [20], and resistance prevalence to this antimicrobial in animal isolates is low [21], although some authors considered this finding worrying [22].

*Enterobacteriaceae* are common inhabitants of the intestinal tract of animals and humans. One of the key mechanisms in the resistance of *Enterobacteriaceae* to antibiotics is the production of β-lactamases [23]. Especially of concern are the extended-spectrum β-lactamases (ESBL), which can inactivate the majority of the β-lactams except for carbapenems and cephamycins [24]. Moreover, resistance determinants of ESBLs are usually located on plasmids [25], and thus can easily be responsible for horizontal gene transfer. On their behalf, extended-spectrum β-lactamases producing *Escherichia coli* have been detected in farm animals, including swine [26]. As food producing animals could be a reservoir of ESBL producers for humans, there is special concern about their prevalence [14].

Furthermore, carbapenemases are the most powerful β-lactamases. These enzymes can hydrolyse almost all available β-lactam antimicrobials including carbapenems [27]. Carbapenemase-producing genes are primarily plasmid-mediated and co-resistance is an important issue to consider [28]. The emerging carbapenem resistance is considered critical since carbapenems are used in human medicine to treat severe infections caused by Gram-negative bacteria [28,29]. Although the use of carbapenems is not allowed in farm animals [29], carbapenem-resistant *Enterobacteriaceae* have been found in food-producing animals [14,24,30].

Moreover, the degree of antibiotic resistance in food-producing animals has been correlated with antibiotic usage, since antibiotic administration can act as a selective pressure for resistant bacteria [31,32]. Tetracyclines and enrofloxacin are antimicrobials used for the treatment of infectious diseases in livestock. Tetracyclines are used to a great extent in animal infections treatment [33]. These antibiotics can play a role in the selection of resistant enterococci since tetracycline-resistant enterococci are often resistant to other antimicrobial agents, such as vancomycin [19]. Enterococci can acquire resistance to quinolones and tetracyclines [19]. Enrofloxacin is a fluoroquinolone used for the treatment of a varied range of diseases in veterinary medicine, such as respiratory and gastrointestinal infections in pigs [34]. Since enrofloxacin can affect intestinal commensal microbiota, there is a special concern about their influence on the selection of resistant bacteria [34].

Thus, the aim of the present work was to evaluate the impact of oxytetracycline or enrofloxacin treatment on vancomycin-resistant enterococci, extended spectrum β-lactamase and carbapenemase-producing *Enterobacteriaceae* in pigs.

## 2. Materials and Methods

### 2.1. Experimental Design

A total of 26 piglets were received from a swine farm on the 5th of September of 2018 in the experimental facilities of the Faculty of Veterinary (Zaragoza, Spain). They were already vaccinated with *Mycoplasma hyopneumoniae* and porcine circovirus type 2 upon arrival. Preventive treatment for coccidia consisting of 0.4 mL/kg of toltrazuril was administered orally to piglets at 3 days of age. No antibiotic treatment was given to the piglets in this period. They were housed in a box that had previously been disinfected and received a sanitary break. They were kept isolated from other animals. The average age of piglets was 28–30 days and mean weight was 43.00 ± 12.79 kg at treatment onset. Each animal was identified with a numbered ear tag. In addition, they were ad-libitum administered an antibiotic-free feed (ARS Alendi, S.A., Huesca, Spain), and water was provided from a separated, controlled water circuit.

The experiment started after an acclimatization period of 40 days. After this time, piglets were divided into three groups and assigned to different pens depending on the antimicrobial treatment given. Group 1 was treated with enrofloxacin (12 piglets: six females and six males), group 2 with oxytetracycline (10 piglets: five females and five males) and group 3 did not receive any treatment (control group) (four piglets: two females and two males).

The following treatments were given to healthy animals under the supervision of qualified veterinarians: Enrofloxacin (100 mg/mL solution) was administered following the treatment guidelines normally used in swine, consisting of a dose of 7.5 mL/kg by intramuscular injection into neck muscles. This treatment consisted of two doses in 48 h, administered alternately on both neck parts of each animal according to its weight. Samples were obtained over 7 days after the administration of the antibiotic. Oxytetracycline was administered according to the guidelines recommended for pigs, consisting of a single dose of 30 mg/kg by intramuscular injection into the neck muscles. Samples were obtained 7 and 19 days after the administration of enrofloxacin and oxytetracycline, respectively. Time 0 of the experiment matched with the last administration of the antibiotic. Samples were collected from the control group on days 0 and 14.

### 2.2. Sampling

Samples were collected from the genital system (vaginal or preputial mucosa) after cleaning the area with a povidone-iodine solution at 10%, aided by a plastic speculum and using a sterile swab. For rectal samples, the area was cleaned in the same way and the swab was introduced in the rectum, using rotational movement to take the sample. All samples were immediately transported to the laboratory and kept at −80 °C until analysis.

The number of animals sampled for each group and day is shown in Table 1. This study was carried out with the animals used in a previous study to evaluate the detection of antibiotics administered in meat and blood [35]. In that study, after antibiotic administration, animals were slaughtered at pre-set intervals within the withdrawal period. For that reason, the number of animals sampled was higher on day 0 compared to the other sampling days. On other hand, in that study, the initial number of animals treated with enrofloxacin was higher than those treated with oxytetracycline. The number of treated animals sampled was six, except on day 0. On day 6, only data of five animals treated with oxytetracycline were shown due to technical problems with one of the samples. The number of animals in the control group was four and samples were only taken on days 0 and 14, due to the complexity of handling such a high number of animals in the veterinary facilities for the previous work [35]. A total of 84 samples were taken from animals treated with enrofloxacin (12 piglets: 42 samples from rectum and 42 samples from the genital system) and 78 from animals treated with oxytetracycline (10 piglets: 39 samples from rectum and 39 samples from the genital system). A total of eight samples were taken from the control group (two piglets: four samples from the rectum and four samples from the genital system).

### 2.3. Bacterial Isolation and Identification

Before microbiological analysis, samples were defrosted. Pre-enrichment was carried out in tubes containing 5.0 mL of Brain Heart Infusion (BHI) broth (Oxoid, Thermo Fisher Scientific, Basingstoke, UK), and incubated at 37 °C for 24 h. After the incubation period, the samples were plated with the streak plate method in three selective chromogenic media: CHROMID^®^ VRE, CHROMID^®^ ESBL and CHROMID^®^ CARBA (BioMérieux, Marcy l’Etoile, France). These media were used to select vancomycin resistant enterococci, ESBL-producing *Enterobacteriaceae* and carbapenemase-producing *Enterobacteriaceae*, respectively. Plates were incubated at 37 °C for 24 h under aerobic conditions.

CHROMID^®^ VRE medium contains a mixture of antibiotics, including vancomycin, which allows the growth of *E. faecium* and *E. faecalis* resistant to this antibiotic. In addition, the chromogenic components provide a preliminary rapid identification of *E. faecium* and *E. faecalis* by the coloration of colonies. CHROMID^®^ ESBL medium contains a mixture of antibiotics, including cefpodoxime, which is the marker of choice for the ESBL resistance mechanism. In addition, the chromogenic components provide a preliminary rapid identification of suspicious ESBL-producing *Enterobacteriaceae* strains by the coloration of colonies including *E. coli*, *Klebsiella*, among others. CHROMID^®^ CARBA medium contains a mixture of antibiotics, which allows the selective growth of carbapenemase-producing *Enterobacteriaceae.* In addition, the chromogenic components provide a preliminary rapid identification of suspicious carbapenemase-producing *Enterobacteriaceae* strains, including *E. coli* and *Klebsiella*, among others.

Isolates were identified by the colour, according to manufacturer instructions. Bacterial species were confirmed by VITEK^®^2 compact (BioMérieux, Marcy l’Etoile, France) in the case of enrofloxacin treated animal isolates and MALDITOF^®^ Biotyper (Bruker, Billerica, MA, USA) in the case of control and tetracycline treated animal isolates. These identification methods are considered reliable for bacteria identification, including *E. faecalis*, *E. faecium*, *E. coli* and *K. pneumoniae* [36,37].

The percentage of animals in which vancomycin resistant enterococci, (ESBL)-producing *Enterobacteriaceae* and carbapenemase-producing *Enterobacteriaceae* was calculated considering the animals harbouring at least one isolate of vancomycin resistant enterococci, ESBL, or carbapenemase-producers over the total number of animals studied.

### 2.4. Statistical Analysis

Data obtained were analysed and submitted to Chi-Square test for comparison of frequencies using SPSS version 26 software (IBM SPSS Statistics). Differences were considered significant if *p* < 0.05.

### 2.5. Ethical Considerations

This work was included in the project “Development of a pioneering self-control solution in live animals to minimize the presence of antibiotic residues in the food chain of the Spain-France cross-border area (POCTEFA-TESTACOS)”, approved by the Ethical Advisory Commission for Animal Experimentation of the University of Zaragoza, reference number PI58/17. The study was carried out in accordance with the ARRIVE (Animal Research: Reporting of In Vivo Experiments) initiative and was handled and used in accordance with the Spanish Animal Protection Policy RD 53/2013 [38], which complies with the European Union Directive 2010/63 [39] on the protection of animals used for experimental and other scientific purposes.

## 3. Results

**Figure 1:**
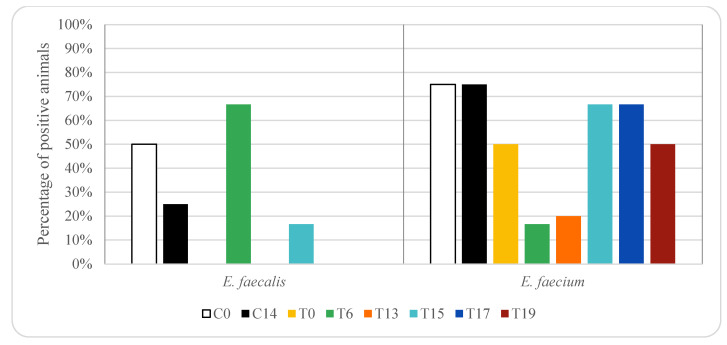
Effect of oxytetracycline treatment on vancomycin-resistant enterococci in pig rectal samples. For group descriptions C0, C14, T0, T6, T13, T15, T17 and T19: see Table 1.

**Figure 2:**
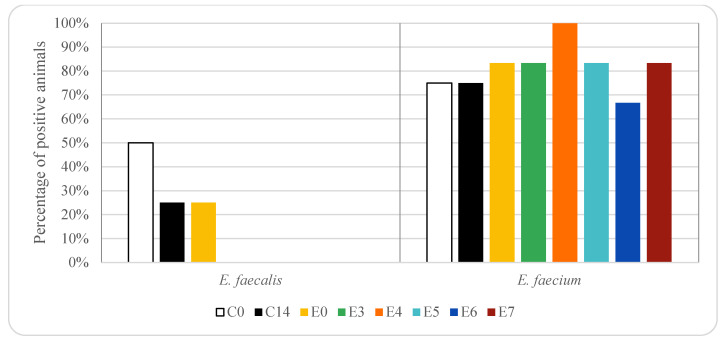
Effect of enrofloxacin treatment on vancomycin-resistant enterococci in pig rectal samples. For group descriptions C0, C14, E0, E3, E4, E5, E6, E7: see Table 1.

Figure 1 and Figure 2 show the percentage of animals in which vancomycin-resistant enterococci (VRE) isolates were found in rectal samples from pigs treated with oxytetracycline and enrofloxacin, respectively. In the control group, without any antibiotic treatment, vancomycin-resistant *E. faecium* was detected in 75% of the animals on days 0 and 14 (three piglets), while *E. faecalis* was detected in 50% (two piglets) and 25% (one piglet) on days 0 and 14, respectively. In animals treated with oxytetracycline, vancomycin-resistant *E. faecium* was detected in 50% of the animals on day 0 (5 piglets), while *E. faecalis* was not detected in any animal (0%). On days 15, 17 and 19 vancomycin-resistant *E. faecium* was detected in 50–67% of the animals (between three and four piglets). On days 17 and 19 vancomycin-resistant *E. faecalis* was not detected in any sample. However, on days 6 and 15 *E. faecalis* was detected in 67% (four piglets) and 17% (one piglet) of the animals, respectively.

In animals treated with enrofloxacin, vancomycin resistant *E. faecium* was detected in 83% of the animals on days 0 and 7 and in 100% on day 4 (10, five and six piglets, respectively). Vancomycin-resistant *E. faecalis* was only detected in 25% of the animals on day 0 (three piglets), corresponding to animals in which *E. faecium* was also isolated. On days 3 to 7 Vancomycin resistant *E. faecalis* was not detected in any animal.

Significant differences (*p* < 0.05) in the prevalence of vancomycin resistant *E. faecium* and *E. faecalis* were found among the three groups of animals studied, and thus, depended on antibiotic treatment.

**Figure 3:**
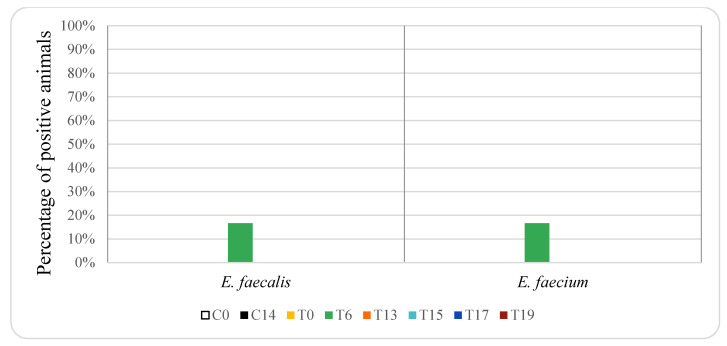
Effect of oxytetracycline treatment on vancomycin-resistant enterococci in pig genital samples. For group descriptions C0, C14, T0, T6, T13, T15, T17 and T19: see Table 1.

**Figure 4:**
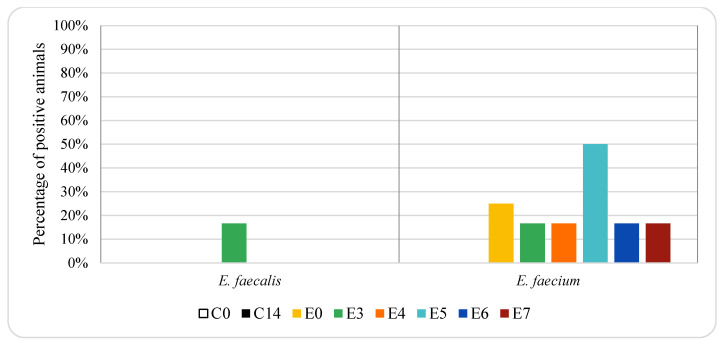
Effect of enrofloxacin treatment on vancomycin-resistant enterococci in pig genital samples. For group descriptions C0, C14, E0, E3, E4, E5, E6, E7: see Table 1.

Figure 3 and Figure 4 show the percentage of animals in which VRE isolates were found in genital samples from pigs treated with oxytetracycline and enrofloxacin, respectively. In the genital samples, *E. faecalis* and *E. faecium* resistant to vancomycin were not detected in control animals. In the animals treated with oxytetracycline, *E. faecalis* and *E. faecium* resistant to vancomycin were only detected on day 6 after treatment (17% *E. faecalis* and 17% *E. faecium*, one piglet). In the animals treated with enrofloxacin, *E. faecium* resistant to vancomycin was detected in 17–50% of the animals (between one and three piglets), depending on the sampling day. No *E. faecalis* resistant to vancomycin was detected in genital samples from animals treated with enrofloxacin, except on day 3, when both *E. faecalis* and *E. faecium* were isolated from 17% of the animals (one piglet). Significant differences (*p* < 0.05) in the prevalence of vancomycin resistant *E. faecium* were found in genital samples among the three groups of animals studied, and thus depended on antibiotic treatment.

**Figure 5:**
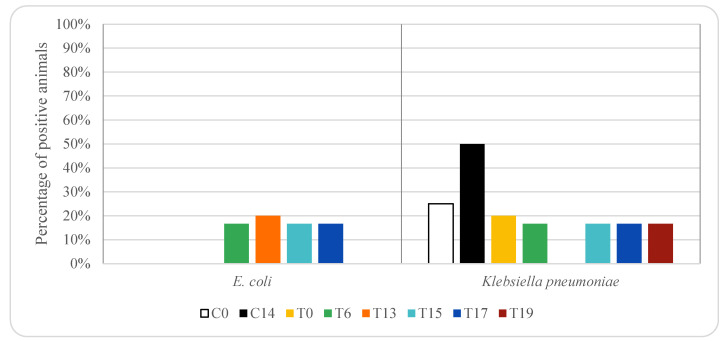
Effect of oxytetracycline treatment on ESBL-producing *E. coli* and *K. pneumoniae* in pig rectal samples. For group descriptions, C0, C14, T0, T6, T13, T15, T17 and T19: see Table 1.

**Figure 6:**
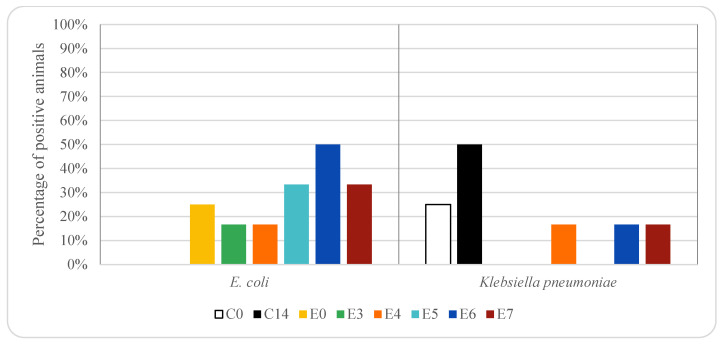
Effect of enrofloxacin treatment on ESBL-producing *E. coli* and *K. pneumoniae* in pig rectal samples. For group descriptions C0, C14, E0, E3, E4, E5, E6, E7: see Table 1.

Figure 5 and Figure 6 show the percentage of animals in which ESBL-producing *E. coli* and *K. pneumoniae* isolates were found in rectal samples from pigs treated with oxytetracycline and enrofloxacin, respectively. ESBL-producing *E. coli* was not detected in rectal samples taken from animals treated with oxytetracycline on days 0 and 19. However, on days 6 to 17, it was isolated among 17–20% of the animals treated with oxytetracycline (1 piglet). In animals treated with enrofloxacin, ESBL producing *E. coli* was detected in 25% of the animals on day 0 (three piglets), on days 3 and 4 was observed in 17% of the animals (1 piglet) and on days 5–7 was among 33 and 50% (between 2 and 3 piglets). ESBL-producing *E. coli* was not detected in rectal samples taken from control animals, not receiving any antimicrobial treatment. Significant differences (*p* < 0.05) in the prevalence of ESBL-producing *E. coli* were found in rectal samples among the three groups of animals studied, and thus depended on antibiotic treatment.

ESBL-producing *K. pneumoniae* was detected in 17–20% of the rectal samples taken from animals treated with oxytetracycline (between one and two piglets), except on day 13 (0%). In animals treated with enrofloxacin, ESBL producing *K. pneumoniae* was 0% on days 0, 3 and 5, while on days 4, 6 and 7 the percentage was 17% (one piglet). ESBL-producing *K. pneumoniae* was detected in control animals, animals that did not receive any treatment, 25% on day 0 (1 piglet) and 50% on day 14 (2 piglets).

**Figure 7:**
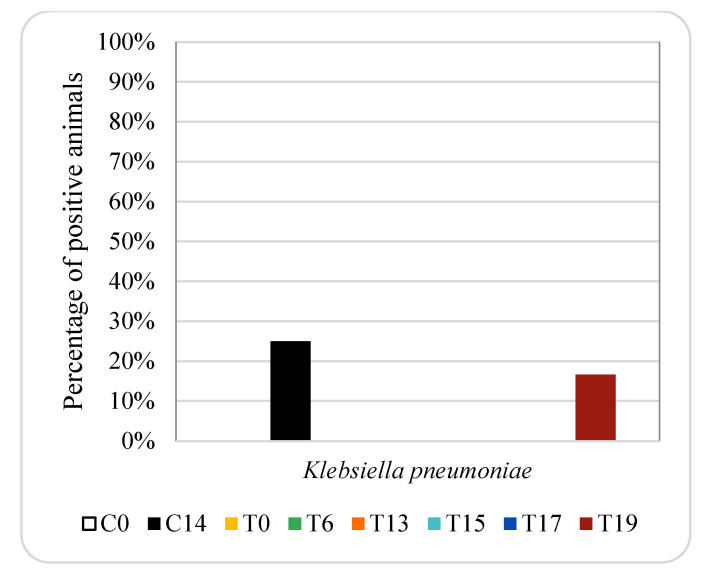
Effect of oxytetracycline treatment on ESBL-producing *K. pneumoniae* in pig genital samples. For group descriptions, C0, C14, T0, T6, T13, T15, T17 and T19: see Table 1.

**Figure 8:**
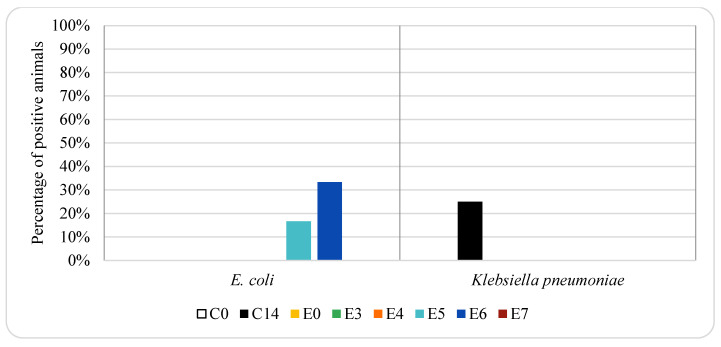
Effect of enrofloxacin treatment on ESBL-producing *E. coli* and *K. pneumoniae* in pig genital samples. For group descriptions C0, C14, E0, E3, E4, E5, E6, E7: see Table 1.

Figure 7 and Figure 8 show the percentage of animals in which ESBL-producing *E. coli* and *K. pneumoniae* isolates were found in genital samples from pigs treated with oxytetracycline and enrofloxacin, respectively. ESBL-producing *E. coli* was not detected either in genital samples taken from animals treated with oxytetracycline or in samples taken from the control group. However, it was detected in 17% and 33% of the animals treated with enrofloxacin on days 5 and 6, respectively (one and two piglets, respectively).

ESBL-producing *K. pneumoniae* was only detected in the 17% of genital samples collected from animals treated with oxytetracycline on day 19 (one piglet), while it was not detected in any animal treated with enrofloxacin. In the control group, it was not detected any positive animal on day 0, while 25% were positive on day 14 (one piglet).

**Figure 9:**
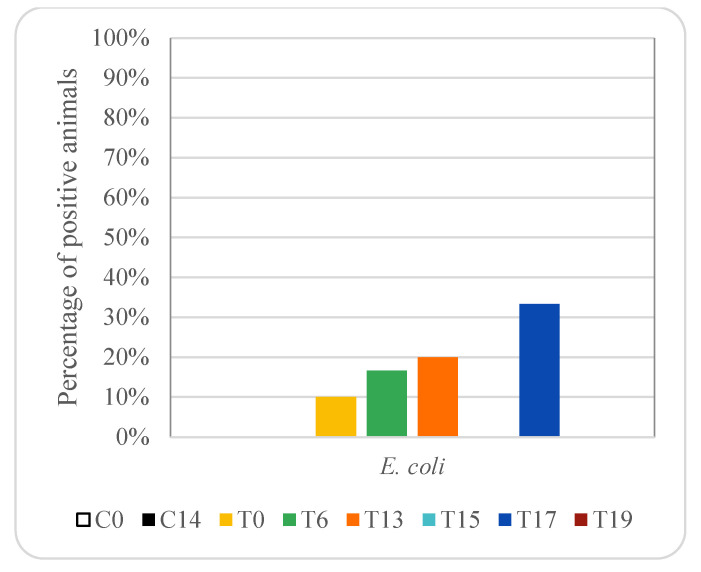
Effect of oxytetracycline treatment on carbapenemase-producing *E. coli* in pig rectal samples. For group descriptions, C0, C14, T0, T6, T13, T15, T17 and T19: see Table 1.

In the animals treated with enrofloxacin, no carbapenemase producers were detected in any sample. However, in animals treated with oxytetracycline, carbapenemase *E. coli* producers were detected in rectum samples on days 0, 6, 13 and 17, with percentages between 10 and 33% (between one and two piglets) (Figure 9), while carbapenemase *K. pneumoniae* producers were not detected in any animal treated with oxytetracycline. Carbapenemase producers were detected neither in rectal samples nor in genital samples from control animals, not treated with antibiotics. Significant differences (*p* < 0.05) in the prevalence of carbapenemase *E. coli* producers were found in rectal samples among the three groups of animals studied, and thus depended on antibiotic treatment.

## 4. Discussion

Some studies carried out in pigs show that *Enterococcus* spp. isolated are susceptible to vancomycin [31,40,41,42,43]. However, other authors have reported the presence of VRE in pigs [22,44,45,46]. Aarestrup et al. [44] observed that 17% of *E. faecium* isolates from pigs in Denmark were vancomycin resistant. The occurrence of VRE in pigs has been associated with the use of avoparcin for growth promotion since avoparcin is a vancomycin analog that confers cross-resistance to vancomycin [47]. Although avoparcin was banned in the European Union in 1997 [48] and later in other countries [49,50,51], the presence of VRE in pigs has still been detected in the last decades [22,45,46]. This fact could be related to co-selection with other antimicrobials [17,22,49].

It should be noted that the occurrence of resistant *Enterococcus* strains in food-producing animals depends on the geographical region since in some areas a high use of antibacterial agents has been observed [31,52,53]. In the present study, vancomycin-resistant enterococci were isolated both in rectal samples from control animals and those treated with oxytetracycline or enrofloxacin. Several authors have reported that a high percentage of enterococci isolated from pigs are resistant to tetracyclines and fluoroquinolones [31,43]. A higher number of vancomycin-resistant *E. faecium* were recovered compared to *E. faecalis*. This finding could be explained since *E. faecium* is more prevalent in pig than *E. faecalis* [32,41,42,43,44]. Prevalence of 45.7% and 12.9% of *E. faecium* and *E. faecalis* have been reported in pigs, respectively [42]. In the control group, the percentage of rectal samples with vancomycin-resistant *E. faecium* was higher than *E. faecalis*. In the pigs treated with enrofloxacin vancomycin resistant *E. faecium* was found in a higher percentage of animals than in the control group and remained above 80% on day 7 (above five piglets). These results can be explained since the persistence of VRE is suggested to be maintained by co-selection, by the use of other antibiotics [17,45,54]. However, in animals treated with oxytetracycline, vancomycin-resistant *E. faecium* was found in a lower percentage of animals compared to the control group. In the case of vancomycin-resistant *E. faecalis* higher percentage of positive animals was found in the control group than in those treated with enrofloxacin or oxytetracycline, except on day 6 after treatment with oxytetracycline, when a percentage above 60% was observed (above 4 piglets). In contrast, Nowakiewicz et al. [43] observed that in a farm in which only oxytetracycline was used, a high percentage of isolated enterococci were susceptible to most of the antimicrobial agents including vancomycin. The high percentage of VRE found in the present work could be explained by the use of specific media to isolate VRE, since VRE could be at a lower level than susceptible enterococci and could be not isolated if a non-selective media is used [42]. In fact, some authors did not detect VRE in pig faecal samples when no selective media were used, but when media were supplemented with vancomycin, VRE were detected [42]. Thus, the isolation media used could explain some of the discrepancies in the prevalence of VRE found in the bibliography [22].

In the present work, a higher percentage of vancomycin-resistant *E. faecium* were found in rectal samples from animals treated with enrofloxacin compared to those treated with oxytetracycline, while a higher percentage of vancomycin-resistance *E. faecalis* was found in rectal samples from animals treated with oxytetracycline compared to those treated with enrofloxacin. These results could be explained by the different susceptibility of enterococci species to these antimicrobials. In fact, Novais et al. [16] reported that *E. faecium* was more often resistant to fluoroquinolones, and *E. faecalis* to tetracyclines. Even more, some authors have only found resistance to fluoroquinolones in *E. faecalis* strains [43].

VRE was not isolated from genital samples in the control group. A lower percentage of VRE was observed in the genital samples in those animals treated with enrofloxacin or oxytetracycline compared to the respective faecal samples. This finding could be explained since enterococci are common inhabitants of the pig intestinal bacteria [17,51]. According to Novais et al. [16], the pig farm environment has an underestimated potential role in the transmission of multidrug resistant *Enterococcus* spp. to animals and, probably, to humans. The contact of pigs with multidrug resistant *Enterococcus* spp. by different routes (air, rooms, feed, dust, etc.) could decrease the impact of restrictive antibiotic use and underline the need of adopting additional control measures. Moreover, oxytetracycline is eliminated in urine (60%) and faeces (40%) [55]. After intramuscular administration of oxytetracycline, an exponential decay has been reported, with its half-life in blood being 3.59 days [35]. Enrofloxacin is mainly excreted via urine and small amounts in faeces [56]. Faster depletion of enrofloxacin compared to oxytetracycline, with a half-life of 1.90 days in blood, has been described [35]. These differences between enrofloxacin and oxytetracycline could affect the surviving bacteria, including VRE.

Moreover, there is a great concern about ESBL and carbapenemase-producing *Enterobacteriaceae* occurring in food-producing animals, since they may constitute a public-health risk [14,57]. Some studies have suggested that ESBL-producing *E. coli* can spread from livestock to humans [58,59]. Besides, the presence of ESBL-producing *E. coli* in swine has been documented by several studies worldwide [8,9,22,24,26,60,61,62,63,64]. High rates of ESBL-producing *E. coli* (up to 70%) have been reported in swine [24,58,59]. Additionally, Graesboll et al. [57] found ESBL-producing coliforms in all the farms evaluated, with 20% of the pigs positive. In the present work, ESBL-producing *E. coli* was not detected in rectal samples from control animals. However, it was detected in 17–20% of animals treated with oxytetracycline on days 6 to 17 (one piglet) and 17–50% of the animals treated with enrofloxacin (between one and three piglets). The percentage of positive animals was higher in pigs treated with enrofloxacin than in those treated with oxytetracycline. The higher prevalence of ESBL-producing *Enterobacteriaceae* when antimicrobials are used has been also reported by other authors. Fournier et al. [65] studied the presence of ESBL-producing *Enterobacteriaceae* in pig rectal samples from two farms, one using antibiotics and the other without antibiotics. These authors reported that the ESBL-producing *Enterobacteriaceae* prevalence was 86% in the farm using antibiotics, while the prevalence in the farm in which antibiotics were not used was 55%. These authors pointed out that there is a link between selective antibiotic pressure and the corresponding resistance rate. They also reported that in the farm using antibiotics, 92% of ESBL producers were resistant to tetracycline, while in the farm where antibiotics were not used co-resistances were lower, with only 44% of ESBL producers resistant to tetracyclines. [65]. It should be noted that some studies show that in pig farms with low antibiotics usage a high percentage of animals are colonised by ESBL-producing *E. coli* [9]. As it is shown in the present study, other authors observed that not all the animals from the same farm were colonised with ESBL producers [22].

Moreover, ESBL producers have been associated with resistance to non-β-lactam antimicrobials, such as fluoroquinolones and tetracyclines, which are often used to treat diseases on pigs [22,24,66,67,68]. Galler et al. [22] isolated ESBL-producing *E. coli* in 46.6% of the swine Austrian farms studied (7 of 15). All isolates were susceptible to fluoroquinolones, while high resistance rates were observed to tetracyclines (73.3%). Additionally, Fournier et al. [9] found low rates of co-resistance to fluoroquinolones among ESBL-producing isolates from pigs. In contrast, Picozzi et al. [66] pointed out that ESBL-producing strains from pigs often presented cross-resistance to quinolones. On their behalf, Liu et al. [67] reported that all ESBL producers isolated from swine exhibited a multidrug resistance phenotype, and more than 90% of them were resistant to tetracycline and enrofloxacin.

Some studies have associated the presence of ESBL-producing *E. coli* with the selective pressure induced by the use of antibiotics in animals [68]. This finding could be linked to the one of De Koster et al. [64], who isolated ESBL-producing *E. coli* in pig faeces from farms with high antibiotic use. However, other studies have shown a high prevalence of ESBL-producing *E. coli* in farm animals with low use of antibiotics (up to 50%) [69,70]. In consequence, other factors apart from antibiotic usages, such as farm conditions, farm environment, farm hygiene and contact with humans could be affecting the presence of ESBL-producing *E. coli* in livestock [24,64,71,72,73]. For instance, Tamta et al. [24] reported that piglets and pig farm workers were a potential source of ESBL-producing *E. coli.* These authors associated the high prevalence of ESBL producing *E. coli* isolates in piglets (44.4%,) and farmworkers (90.5%) to the use of the selective medium for detecting resistant *E. coli* isolates or the use of β-lactam and cephalosporin antibiotics on the farms studied. Other authors have also reported a high percentage of ESBL-producing *E. coli* using selective media [74,75]. In the present work, CHROMID ESBL medium was used to isolate ESBL-producing *E. coli*. This medium allows detecting ESBL producers when they are present at low levels, especially *E. coli*, which is one of the most frequent ESBL producers [76,77,78,79].

As stated above, the use of antibiotics in food-producing animals may select bacteria resistant to them. Moreover, the antimicrobial treatment affects both the targeted pathogen and the commensal bacteria. Since some amount of administrated antibiotics end up in the intestines [80], the intestinal tract of animals acts as an important reservoir for the selection of antibiotic resistance [64,81]. Thus, the formation and selection of resistant strains in the intestinal commensal bacteria could play an important role in the spread of resistant bacteria [82]. Several studies have shown that tetracycline resistant commensal *E. coli* bacteria from livestock are often resistant to other antimicrobials, indicating co-selection [83,84,85]. Jensen et al. [85] reported that the usage of tetracycline in pig farms can promote resistance to critically important antimicrobials. Tetracycline resistance is often found in ESBL-producing isolates and transmitted with ESBL containing plasmids [86]. In contrast, Gruel et al. [33] reported that the use of tetracycline is not correlated to ESBL-producing *E. coli*. Although tetracycline is not listed as critically important for human treatment [20], there is a great concern since it can promote resistance to other antimicrobials.

Furthermore, the way of administration of antimicrobials might also be a critical factor to consider when talking about selective pressure for resistant bacteria. Intramuscular administration of antibiotics is considered to have lower influence on intestinal microbiota than oral administration, since it does not require absorption from the gut [34]. Nevertheless, although renal excretion has been reported as the main excretion mechanism of enrofloxacin after intramuscular administration of enrofloxacin [86], high faecal concentrations of this antibiotic have been found in pigs [34]. These high enrofloxacin faecal levels could affect the faecal microbiota, especially Gram-negative bacteria including *E. coli*, as it has been documented that enrofloxacin is effective against these bacteria [87]. On the other hand enrofloxacins can reach high concentrations in the gut because they are partially excreted in the bile acid [88]. Moreover, intestinal efflux transporters may transport enrofloxacin into the gut lumen [89]. Thus, enrofloxacin can disrupt the gut commensal bacteria, even when treatment is by intramuscular injection [90]. Enrofloxacin can influence the population dynamics of enteric bacteria and may select for resistance [91]. Besides, it has been observed that intramuscular enrofloxacin treatment reduces the faecal *E. coli* wild type population [34,92], and some authors have observed that at the end of enrofloxacin treatment only non-wild type *E. coli* isolates are found in faeces [34]. Moreover, Béraud et al. [92] reported that the intramuscular administration of enrofloxacin reduced the faecal *E. coli* counts from 3.79 log cfu/g to counts below 2 log cfu/g. Afterward, these authors observed a regrowth of *E. coli*, and these *E. coli* isolates recovered from pigs with intramuscular administration of enrofloxacin in the regrowth stage, were resistant to enrofloxacin and other antibiotics. Römer et al. [93] also observed that intramuscular administration of enrofloxacin in pigs caused an important reduction of the susceptible intestinal *E. coli* population, in favour of resistant *E. coli.* These authors pointed out that the intramuscular administration of enrofloxacin reduced the susceptible intestinal *E. coli* population, which was replaced by enrofloxacin resistant strains, but also control pigs were affected, maybe due to the transferability of strains through the environment. Wiuff et al. [94] also observed an increase of resistance in *E. coli* from the gut of pigs after intramuscular administration of enrofloxacin. Since intramuscularly administered enrofloxacin may exert selective pressure on the intestinal microbiota, including *E. coli*, there is a risk of resistance selection [92]. Besides, some of the studies found in the bibliography administered ciprofloxacin instead of enrofloxacin. It should be taken into account that after administration in pigs, enrofloxacin partially metabolizes into ciprofloxacin [95]. The metabolic conversion of enrofloxacin to ciprofloxacin is 51.5% in healthy pigs [95]. It should be noted that fluoroquinolones are important antibiotics for the treatment of infections in humans and they have been categorised as “highest priority critically important antimicrobials” [20].

On the other hand, despite rational use of antimicrobials, the rate of ESBL-producing *E. coli* in livestock, although moderate, is of concern [33]. Fewer data are available on ESBL-producing *K. pneumoniae*, since most of the studies are related to ESBL-producing *E. coli*. On the other hand, *K. pneumoniae* is present in lower numbers compared to *E. coli* [64]. De Koster et al. [64] isolated resistant *Enterobacteriaceae* from poultry and pig, 91.4% were identified as *E. coli*, whereas only 1.78% were identified as *K. pneumoniae*. ESBL-producing *K. pneumoniae* has been isolated in meat [96] and in pigs [97]. Since colonisation with *E. coli* and other *Enterobacteriaceae* occurs in the digestive tract, a higher percentage of positive animals were found in rectal samples than in genital samples [10]. Moreover, the presence of antibiotics in faeces could select ESBL and carbapenemase-producing *E. coli* [34].

Several authors have not detected carbapenemase-producing *Enterobacteriaceae* in pigs [9,65]. However, some studies have found carbapenemase-producing *E. coli* in pigs [14,24,75,98,99]. Carbapenemase producing *E. coli* have been isolated from the environment of swine farms in the USA [75]. Additionally, carbapenemase-producing *Enterobacteriaceae* have been reported in pig farms in Germany [100]. According to Köck et al. [30] the prevalence of carbapenemase-producing *Enterobacteriaceae* in farm animals is low in Europe (<1%), whereas a higher prevalence has been observed in China, India and Algeria. Specifically, the prevalence of carbapenemase-producing *E. coli* in pigs is low, since carbapenems are not used in food-producing animal treatment [98]. Indeed, Fournier et al. [9,65] did not detect carbapenemase-producing *Enterobacteriaceae* in pigs from farms using or not using antibiotics. Related to this varying occurrence, it has been suggested that carbapenem resistant *E. coli* in pigs could originate from the human contact environment [24]. According to Dandachi et al. [10], the emergence of carbapenemase-producers in livestock is related to the co-selective pressure by the usage of non-β-lactams antibiotics. It should be noted that the spread of carbapenemase-producing *Enterobacteriaceae* is of great concern since they are multidrug-resistant [27] and their presence in animals could constitute a reservoir and be a risk for human health [14]. As an example, carbapenemase producers are often co-resistant to non-β-lactam antibiotics including tetracyclines and fluoroquinolones [101].

Data presented in this work show that carbapenemase-producing *Enterobacteriaceae* were not detected in any rectal sample taken from control animals or those treated with enrofloxacin. However, carbapenemase *E. coli* producers were detected on days 0, 6, 13 and 17, with percentages between 10 and 33% in animals treated with oxytetracycline (between one and two piglets). These results suggest that tetracycline exposure could influence the occurrence of carbapenemase-producing *E. coli.* A selective medium, CHROMID CARBA, was used for the detection of carbapenemase-producing *Enterobacteriaceae*; since these bacteria could be in low numbers, it is possible that the use of non-specific media fails to detect them [102]. Moreover, carbapenemase-producing *E. coli* were not isolated in genital samples, and carbapenemase-producing *K. pneumoniae* were not isolated either in faecal samples or genital samples. There are few data available on carbapenemase-producing *K. pneumoniae*. Only some studies have found carbapenemase-producing *K. pneumoniae* in poultry [103,104].

It should be noted that in the present study samples were collected and immediately frozen at −80 °C. As pointed out by other authors, the immediate processing of faecal samples after collection is not always technically or economically feasible with freezing being the most common method of preservation [105,106]. The survival and diversity of microbial populations in faecal samples after freezing and storage at −80 °C have been evaluated by several studies [105,107,108]. Masters et al. [105] studied the survival and diversity of *E. coli* and enterococci populations in faecal samples of animal origin (including pig) after storage at either −20 or −80 °C for 30 days. These authors reported that the numbers of enterococci were similar in fresh and frozen faecal pig samples. The number and the distribution of *E. coli* and enterococci assigned to different biochemical phenotypes in fresh samples did not vary significantly from those stored at −80 °C [105]. Furthermore, the population structure of *E. coli* and enterococci did not change significantly after storage at −80 °C [105]. Similar findings were reported by Tedjo et al. [107], who did not observe significant changes in the overall microbiota composition between frozen faecal samples at −80 °C and samples stored at room temperature or 4 °C for 24 h. According to Deschamps et al. [108], freezing at −80 °C without cryoprotectant was the most efficient method for faecal preservation considering both stabilization time of microbial profiles and metabolic activities.

This study was carried out with three groups of animals: group 1 treated with enrofloxacin (N = 12), group 2 treated with oxytetracycline (N = 10) and group 3 that did not receive any treatment (control group) (N = 4). The three groups were kept separated in the experimental facilities of the Faculty of Veterinary. They were maintained in the same conditions, then the effect of the farm environment, farm hygiene, and personal hygiene of farmworkers were the same in the three groups. The effect of the antibiotic treatments has been compared with the control group. The results obtained show that there are differences between animals treated with antibiotics and those non-treated on VRE, ESBL and carbapenemase-producing *Enterobacteriaceae*. Further works are needed to know the role of antibiotic treatments on antimicrobial resistance. The differences found with other studies could be due to the influence of farm environment, farm hygiene and personal hygiene of farmworkers.

Thus, the degree of antibiotic resistance in food-producing animals has been correlated with antibiotic usage, since antibiotic administration can act as a selective pressure for resistant bacteria [32,33]. Data presented prove that special care should be taken in the slaughter process to avoid the faecal contamination of pig carcasses since *Enterobacteriaceae* and enterococci are normally present in the intestinal tract and could be multi-resistant bacteria [23], resistance tightly linked to selective pressure triggered by antimicrobial treatment. This cross-contamination of animal carcasses may be a food safety risk [42,94] as it might be enhancing the already serious problem of antimicrobial resistance dissemination. On the other hand, dissemination could occur if the environment is contaminated by pig faeces.

## 5. Conclusions

This study highlighted that the use of tetracycline in food-production animals could select ESBL and carbapenemase-producing *E. coli* in the intestinal tract as suggested by the results found in rectal samples; while the use of enrofloxacin could select ESBL-producing *E. coli* in the intestinal tract and in a lesser extent in the genital system. Thus, special care should be taken to avoid faecal contamination of carcasses during slaughter. Additional studies are needed on ESBL-producing *K. pneumoniae*, since data presented in this study pointed to this bacterium being present in pigs. Vancomycin resistant *E. faecium* can be present in faeces from pigs; the treatment with enrofloxacin could increase the percentage of positive animals. The high percentage of animals with the presence of VRE found underlines the relevance of using selective media for the isolation of VRE. It should be noted that in the present study only chromogenic media were used and further works are needed to confirm the results obtained. On the other hand, further research is needed to estimate the magnitude of the effect of antibiotic treatments on antimicrobial resistance and to know the mechanisms involved. Further studies shall be needed to validate the results obtained, considering a more robust and extended experimental design.

## Figures and Tables

**Table 1:** Number of animals sampled for each treatment day.

Treatment	Day	Sample Code	Number of Animals
Control	0	C0	4
	14	C14	4
Oxytetracycline	0	T0	10
	6	T6	6
	13	T13	5
	15	T15	6
	17	T17	6
	19	T19	6
Enrofloxacin	0	E0	12
	3	E3	6
	4	E4	6
	5	E5	6
	6	E6	6
	7	E7	6

## Data Availability

Not applicable.
